# Temporal pathways of change in two randomized controlled trials for depression and harmful drinking in Goa, India

**DOI:** 10.1017/S0033291718003963

**Published:** 2019-01-08

**Authors:** Daisy R. Singla, Steven D. Hollon, Richard Velleman, Benedict Weobong, Abhijit Nadkarni, Christopher G. Fairburn, Bhargav Bhat, Mahesh Gurav, Arpita Anand, Jim McCambridge, Sona Dimidjian, Vikram Patel

**Affiliations:** 1 Department of Psychiatry, University of Toronto and Sinai Health System, Toronto, Canada; 2 Department of Psychology, Vanderbilt University, Nashville, Tennessee, USA; 3 Department of Psychology, University of Bath, Bath, UK; 4 Sangath, Alto Porvorim, Goa, India; 5 Centre for Global Mental Health, Faculty of Epidemiology and Population Health, London School of Hygiene and Tropical Medicine, London, UK; 6 Department of Social & Behavioral Sciences, School of Public Health, University of Ghana, Accra, Ghana; 7 Department of Psychiatry, Warneford Hospital, Oxford, UK; 8 Department of Health Sciences, University of York, York, UK; 9 Department of Psychology and Neuroscience, University of Colorado Boulder, Boulder, Colorado, USA; 10 Department of Global Health & Social Medicine, Harvard Medical School, Cambridge, USA; 11 Department of Global Health and Population, Harvard TH Chan School of Public Health, Boston, MA, USA

**Keywords:** Alcohol use disorders, behavioral activation, counter-change talk, depression, implementation science, mediation, motivational interviewing, process research, psychological treatments

## Abstract

**Background:**

The current study explored the temporal pathways of change within two treatments, the Healthy Activity Program (HAP) for depression and the Counselling for Alcohol Problems (CAP) Program for harmful drinking.

**Methods:**

The study took place in the context of two parallel randomized controlled trials in Goa, India. *N* = 50 random participants who met *a priori* criteria were selected from each treatment trial and examined for potential direct and mediational pathways. In HAP, we examined the predictive roles of therapy quality and patient-reported activation, assessing whether activation mediated the effects of therapy quality on depression (Patient Health Questionnaire-9) outcomes. In CAP, we examined the predictive roles of therapy quality and patient change- and counter-change-talk, assessing whether change- or counter-change-talk mediated the effects of therapy quality on daily alcohol consumption.

**Results:**

In HAP, therapy quality (both general and treatment-specific skills) was associated with patient activation; patient activation but not therapy quality significantly predicted depression outcomes, and patient activation mediated the effects of higher general skills on subsequent clinical outcomes [*a* × *b* = −2.555, 95% confidence interval (CI) −5.811 to −0.142]. In CAP, higher treatment-specific skills, but not general skills, were directly associated with drinking outcomes, and reduced levels of counter-change talk both independently predicted, and mediated the effects of higher general skills on, reduced alcohol consumption (*a* × *b* = −24.515, 95% CI −41.190 to −11.060). Change talk did not predict alcohol consumption and was not correlated with counter-change talk.

**Conclusion:**

These findings suggest that therapy quality in early sessions operated through increased patient activation and reduced counter-change talk to reduce depression and harmful drinking respectively.

## Introduction

Evidence-based psychological treatments (PTs) are recommended as the first line of treatment for depression and alcohol use disorders (AUD) – two leading causes of global burden of mental disorders for women and men respectively (World Health Organization, 2015). Worldwide, brief PTs delivered by non-specialist providers (NSPs) have been successful at treating adult depression and AUDs (Van Ginneken *et al*., 2013; Hoeft *et al*., 2016; Singla *et al*., 2017). Despite their effectiveness, it remains unclear how these treatments achieve their effects. Identifying temporal pathways of how key treatment and patient variables influence clinical outcomes may illuminate how PTs operate, guiding clinicians, and researchers to predict individual patient trajectories and develop more effective interventions (Cook *et al*., 2015; Kraemer, 2016).

The current study explored specific treatment- and patient-level variables that may influence the effectiveness of lay counselor delivered treatments for depression and harmful drinking. Each treatment was the subject of a randomized controlled trial (RCT), delivered in primary care settings in Goa, India. The Healthy Activity Program (HAP) – a brief, culturally adapted version of behavioral activation (BA) treatment for depression (Chowdhary *et al*., 2016) – was found to be more effective than enhanced usual care (EUC) in reducing depressive symptoms and improving remission rates at the primary end-point of 3-months (Patel *et al*., 2017). Similarly, the Counselling for Alcohol Problems (CAP) Program – a brief, motivational interviewing (MI)-informed PT for harmful drinkers (Nadkarni *et al*., 2015) – was found to be more effective than EUC in increasing abstinence and remission rates at 3-months post-enrollment (Nadkarni *et al*., 2017*a*, *b*).

**Fig. 1.**
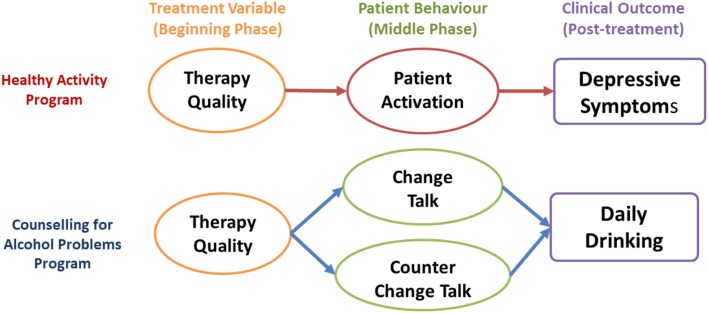
Hypothesized temporal pathways of change.

As per Magill *et al*. (2014, 2015), we defined *treatment variables* as treatment processes or counselor skills that predict patient behaviors or long-term effects. *Patient behaviors* were those enacted by the patient that were potentially associated with the outcomes of interest (i.e. average daily drinking scores and depressive symptoms at 3-months, post enrollment). Fig. 1 shows the study's hypothesized pathways of change in each trial, whereby both treatment-specific and general skills in early sessions would influence the subsequent patient behaviors (patient activation in HAP or change and counterchange talk in CAP) which would, in turn, influence the clinical outcome post-treatment. We also hypothesized that patient behaviors would mediate the effects of therapy quality on clinical outcomes.

### Treatment variable

In both HAP and CAP, our treatment variable was therapy quality – the degree to which the counselors have ‘done the right things well’ (Fairburn and Cooper, 2011). Therapy quality included both general skills (e.g. empathy, collaboration, and expressing warmth) and treatment-specific skills (e.g. activity monitoring, scheduling, and homework completion in HAP and avoiding confrontation, providing affirmations and emphasizing personal choice in CAP; Singla *et al*., 2014). In the depression literature, therapist behaviors account for around 5–10% of unexplained variance in patient outcomes and hold in different therapy models and after controlling for confounding patient variables (Kim *et al*., 2006). Similarly, across the MI literature, growing evidence suggests that high levels of treatment-specific therapy quality (or ‘MI-consistent’ skills) are favorably linked to within-session patient behaviors (Magill *et al*., 2010). A recent RCT demonstrated that higher treatment-specific therapy quality showed better patient outcomes, irrespective of the level of experience of the counselor in treatment delivery (Gaume *et al*., 2014*a*).

### Patient behaviors

In HAP, the patient variable specified by theory was patient-reported activation (patient activation). Patient activation levels have been found to mediate the effects of BA treatments on depression outcomes (Gaynor and Harris, 2008), including the sustained effects seen in the larger HAP effectiveness trial (Weobong *et al*., 2017); but no study to date has examined the potential relations between patient activation, therapy quality and depression outcomes.

In CAP, patient behaviors were change talk and counter-change talk. Both theory and research suggest a positive relationship between MI-consistent skills and patient change talk (Miller and Rose, 2009), and a negative relationship between MI-consistent skills and patient counter-change talk (Moyers *et al*., 2009; Gaume *et al*., 2014*b*; Magill *et al*., 2014; Pace *et al*., 2017). There is inconclusive evidence about whether patient change talk mediates the effects of therapy quality on patient drinking outcomes (Barnett *et al*., 2014). A recent meta-analysis showed that general skills (e.g. empathy, collaboration) among therapists were related to increased treatment-specific skills and to increased patient change talk but not to patient clinical outcomes (Pace *et al*., 2017).

In the current study, and in order to better understand the effectiveness of HAP and CAP treatments, we explored the potentially predictive and mediating roles of treatment and patient behaviors on depression and drinking outcomes at 3-months post-enrollment. Our research questions were:

(1) Does therapy quality (general and treatment-specific skills) predict patient behaviors (patient activation in HAP and change and counter-change talk in CAP) and clinical outcomes (depression severity in HAP and daily alcohol consumption in CAP)?
(2) Do patient behaviors predict clinical outcomes?
(3) Do patient behaviors mediate the effects of therapy quality on clinical outcomes?

## Methods

### Setting

This secondary analysis was conducted within the context of PREMIUM (PRogram for Effective Mental health Interventions in Under-resourced health systeMs), a research program designed to develop and evaluate brief, contextually appropriate PTs for lay counsellor delivery in primary care in Goa, India (Patel *et al*., 2014). Both the HAP and CAP trials were conducted concurrently (with the two treatments being delivered by the same counselors) in 10 primary health centers. HAP patients were aged 18–65 years, with moderately severe to severe depressive symptoms on the Patient Health Questionnaire (PHQ-9; Spitzer *et al*., 1999). CAP patients were male, aged 18–65 years, who were harmful drinkers, defined as scoring 12–19 on the AUDIT (Saunders *et al*., 1993). Only males were included in the CAP trial because the prevalence of AUDs is low in Indian women (Rathod *et al*., 2015). Selection procedures for each trial including inclusion and exclusion criteria, patient characteristics, and outcomes are detailed elsewhere (Nadkarni *et al*., 2017*a*; Patel *et al*., 2017). The manuals for both HAP (http://www.sangath.in/wp-content/uploads/2015/08/Healthy%20Activity%20Program_Manual.pdf) and CAP (http://www.sangath.in/wp-content/uploads/2015/08/Counselling%20for%20Alcohol%20Problems_Manual.pdf) and training website (http://premium.nextgenu.org) can be accessed online. Institutional Review Boards at Sangath, the London School of Hygiene and Tropical Medicine, and the Indian Council of Medical Research provided ethical approval.

### Procedures

#### Samples

Two subsamples were randomly selected from the active treatment arm of each trial. Both samples included treatment arm participants only because relevant variables were not collected in the control group of the larger effectiveness trials. Random sampling was used to ensure that the selection of tapes was not biased. Due to the resource-intensive nature of independently rating transcripts, only a subset of the larger group of eligible participants was used in this secondary analysis; however, our methods met the conditions suggested by other mediation studies including a sample size of 40 or more participants, temporally focused relations between mediator and outcome, independent raters of variables of interest, and examined within randomized designs (MacKinnon and Pirlott, 2015; Lemmens *et al*., 2016). A recent review of mediators in psychological treatment trials for depression (Lemmens *et al*., 2016) recommended 40 or more participants after taking into account both statistical power calculations as well as the average sample sizes in the literature. In HAP, *N* = 50 participants were randomly selected based on their 3-month remission status (25 remitters and 25 non-remitters, where remission is defined as a PHQ-9 score < 10) from a pool of patients who had received at least two treatment sessions in the middle phase of treatment (sessions 3–5, see below). In CAP, *N* = 50 participants were randomly selected based on their 3-month remission status (25 remitters and 25 non-remitters where remission is defined as AUDIT score < 8) from a pool of patients who had received at least two treatment sessions (sessions 1 and 2), both of which were of good audio quality. The rationale was two-fold: (a) to include only those patients exposed to sessions in which core HAP and CAP content was delivered; and (b) to assess the temporal relations (Kazdin, 2007) between therapy quality in the beginning phase (sessions 1–2 in HAP and session 1 in CAP) and patient behaviors in the subsequent session (sessions 3–5 in HAP and session 2 in CAP). No other constraints were applied.

#### The treatments

Lay counselors with no previous experience in mental health care were trained to deliver both HAP and CAP. Details regarding the selection, training, and supervision of the lay counselors can be found elsewhere (Singla *et al*., 2014).

#### Health Activity Program (HAP)

HAP was a six–eight session treatment, delivered in three phases (beginning, session 1; middle, sessions 3–5; and end, session 6–8). It is a BA PT (Dimidjian *et al*., 2011) adapted for the local context (Chowdhary *et al*., 2016). Key strategies include psychoeducation, behavioral assessment, activity monitoring, activity structuring and scheduling, activation of social networks, and problem-solving (Chowdhary *et al*., 2016).

#### Counseling for alcohol problems (CAP)

CAP is a manualized, MI-informed PT with cognitive-behavioral procedures incorporated. It was delivered in three phases (beginning, middle, and end) and culturally adapted for the local context (Nadkarni *et al*., 2015). Key strategies included a detailed assessment with personalized feedback and some psychoeducation (beginning phase), helping the patient to develop problem-solving skills, managing peer pressure, drink refusal and emotional regulation strategies (middle phase), and managing potential relapses by reinforcing key skills (end phase). The therapist's role was to reflect a MI stance while implementing a patient-centered approach to facilitating change that aimed to enhance motivation while simultaneously providing warmth, empathy, and collaboration.

#### Outcomes

The primary outcome for HAP was depressive symptoms severity scores on the PHQ-9 (Spitzer *et al*., 1999) at 3-months post-enrollment as assessed by independent evaluators who were blind to treatment status. This variable was selected over the trial's other primary outcome of remission status because depressive symptoms offered a continuous score whereas remission was defined as a binary variable (PHQ-9 scores < 10). A continuous score is recommended over binary variables in regression to capture adequate variance (MacKinnon *et al*., 2007*b*).

The primary outcome for CAP was mean daily drinking consumption (measured in grams of ethanol) in the 14 days immediately preceding the 3-month assessment, as assessed by independent evaluators who were blind to treatment status, who calculated this consumption using the timeline follow back method (Sobell and Sobell, 1992). Mean daily alcohol consumption was selected as the primary outcome for most of these analyses for two reasons: it is the most widely used outcome in alcohol trials (Bertholet *et al*., 2005); and offers a continuous score (MacKinnon *et al*., 2007*b*).

### Independent variables

#### Treatment variable

*Therapy quality* was assessed by one group of four independent raters using the HAP and CAP Therapy Quality Scale (TQS^†^^1^, Singla *et al*., 2014). The instrument consists of two subscales assessing general and treatment-specific skills (elements prescribed for the therapeutic modality of HAP or CAP). The general skills subscale involves 10 items (which are the same for HAP and CAP); the treatment-specific skills subscales involve up to 15 items, (different for HAP and CAP), and based on the phase-specific session being rated. The mean of each subscale (0–4) was used to independently estimate general and treatment-specific skills respectively. To ensure temporal distinction between therapy quality and patient behaviors, therapy quality was assessed using beginning phase audiotapes (session 1 in CAP and session 1 or 2 in HAP) only. Psychometric properties of TQS ratings were established in a previous study which demonstrated good concurrent validity between counselor-based ratings and expert ratings (Singla *et al*., 2014). While we assumed that treatment-specific skills build upon general therapeutic skills, we assessed the two separately to determine whether they uniquely contributed to hypothesized temporal pathways.

*Patient behaviors* were assessed by a second set of four independent raters who were not involved in the rating of therapy quality.

(1) *Patient activation* was assessed using the PREMIUM Abbreviated Activation Scale (PAAS), which was based on the Behavioral Activation for Depression Scale (Kanter *et al*., 2007). This five-item scale assessed to what extent patients reportedly engaged in healthy activities and accomplished their between-session goals, each rated on a scale of 0–5 (hence the overall PAAS scores could range between 0 and 25). Patient activation was assessed during the middle phase (sessions 3–5). Reliability estimates of PAAS ratings across four separate transcripts indicated strong inter-rater reliability during the training phase (ICC = 0.815) and internal consistency (*α* = 0.885).
(2) *Change talk* and *counter-change talk* were assessed using the Motivational Interviewing Skill Code (MISC), version 1.1 (Glynn and Moyers, 2012). This measure is an observational coding system that categorizes and quantifies patient change language and gives separate frequency counts of change talk and counter-change talk utterances within the rated session (Glynn and Moyers, 2009). We selected the MISC 1.1 because, unlike later versions of the MISC and alternative measures of change-talk (e.g. Motivational Interviewing Treatment Integrity), the MISC assessed our construct of interest (rather than additional therapist behaviors which were captured by the TQS described above), was easy to use, and had shown good predictive validity on relevant clinical outcomes (Lombardi *et al*., 2014). Change talk and counter-change talk were assessed using session 2 (middle phase) audiotapes only. Similar to other studies (Moyers *et al*., 2009; Campbell *et al*., 2010), we calculated separate summed score for change talk and counter-change talk. These four independent raters all received training in the use of the MISC 1.1 over 2 weeks. After three rounds of pilot testing, reliability estimates of MISC ratings across four transcripts among the four independent raters indicated good interrater reliability (ICC = 0.718).

Baseline sample characteristics related to the patient (age, education, marital status, occupation, and PHQ-score) and the counselor or treatment (average session duration, recruitment period) were examined as potential covariates.

### Data collection

There were three groups of independent data collectors involved in this study; all assessors were blind to the ratings by other assessors. First, independent interviewers assessed primary outcomes at the 3-month endpoint. These data were recorded using tablets that were uploaded in real-time to a server with data being reviewed by independent data managers. Second, as described above under the Independent variables section, two separate groups of four counselors were all blind to outcome status and each independently assessed either therapy quality or patient behaviors from audiotaped sessions using the TQS, and the PAAS or MISC respectively. Randomly selected ratings were quality checked by expert clinicians. No counselor rated his or her own sessions.

### Analyses

All analyses were exploratory, using SAS 9.4 and R-studio. First, means and confidence intervals (CIs) for each variable were estimated. Second, Pearson correlations were used to assess the associations among independent variables. If trend or significant relationships were observed (*p* < 0.10), variables were added to the regression models (described next) to account for maximum change in average daily alcohol consumption and for any potential confounding variables. Similar to other mediation studies that apply a lower threshold (Hayes, 2015), we defined significance as *p* < 0.10. Among CAP participants, we used baseline AUDIT scores to account for baseline problem severity (these were significantly related to mean daily alcohol consumption at follow-up, *r* = 0.217, *p* = 0.005) because mean daily alcohol consumption amounts were not collected at baseline.

Next, we used multiple linear regression to run models where the dependent variable was 3-month daily alcohol consumption in CAP or depressive symptoms in HAP. In each study sample, our models estimated, whether: (a) treatment-specific or general skills in the beginning phase predicted 3-month clinical outcomes; (b) treatment-specific or general skills predicted subsequent patient behaviors (patient activation in HAP and change or counter-change talk in CAP) in the middle phase; and, (c) middle-phase patient behaviors predicted subsequent 3-month outcomes, while accounting for treatment-specific or general skills. Variance inflation factor (VIF) was assessed for each independent variable within each model to estimate multicollinearity (VIF ⩾ 5).

If mediation conditions were met^2^, we examined whether (respectively) patient activation or change talk and counter-change talk mediated the effects of therapy quality on mean depressive symptoms in HAP or daily drinking outcomes in CAP using the Monte Carlo Method for Assessing Mediation (MCMAM; Selig and Preacher, 2008). In this approach, a distribution of the indirect effect was used to estimate a CI around the observed value of the indirect effect (MacKinnon *et al*., 2004). MCMAM performs better than the Sobel test and comparably with bootstrap approaches (MacKinnon *et al*., 2002; MacKinnon and Pirlott, 2015) and no direct effect is required of the independent variable (in this case, therapy quality) on the dependent variable (either depressive symptoms or mean daily alcohol consumption at 3-months; MacKinnon *et al*., 2007*a*). In the current study, we computed a 95% CI with 20 000 repetitions.

## Results

Among HAP patients (*N* = 50), 80.0% were female and the sample had an average age of 44.26 years (95% CI 41.21–47.34). The majority were married (72.0%), Hindu (98.0%), homemakers (66.0%), and had completed a minimum of primary level education (80.0%). The patients had received an average of 6.92 sessions (95% CI 6.65–7.19, range 0–8) that lasted an average of 38.93 min (95% CI 37.03–40.84). Mean PHQ-9 scores of 18.66 (95% CI 17.87–19.45) at baseline decreased to 9.34 (95% CI 6.70–8.57) at 3-months post-enrollment.

The CAP patients (*N* = 50) were, on average, 44.06 years of age (95% C 41.16–46.96). Most were married (78.0%), Hindu (88.0%), unskilled manual laborers (66%), and had completed a minimum of primary level education (70.0%). Out of a maximum four sessions, these patients had received an average of 3.06 sessions (95% CI 2.84–3.28, range 0–4) that lasted an average of 40.26 min (95% CI 38.5–42.0). At 3-months post-enrollment, their mean daily drinking was 19.96 g ethanol (95% CI 8.25–31.67) of ethanol.

The two subsamples were similar to the larger samples in terms of demographic characteristics (Nadkarni *et al*., 2017*b*; Patel *et al*., 2017).

### Therapy quality, patient activation, and depression outcomes

The mean beginning phase treatment-specific and general skills scores were 2.45 (95% CI 2.30–2.61) and 2.71 (95% CI 2.60–2.81) respectively and mean PAAS scores were 13.4 (95% CI 12.41–14.35) out of a maximum score of 25. The correlation analyses indicated significant, positive associations between therapy quality treatment-specific skills and patient activation (*r* = 0.303, *p* = 0.034), therapy quality general skills and patient activation (*r* = 0.346, *p* = 0.015) as well as a significant negative association between patient activation and PHQ-9 outcome scores (*r* = −0.458, *p* = 0.0008). These, in turn, suggest medium effect sizes between independent and dependent variables of interest (Cohen, 1988). Recruitment period, baseline depression and treatment dose also were also significantly related to one or more key variables and were therefore added to the regression models.

Table 1 shows the results of the regression and mediation analyses. Treatment-specific and general therapy quality scores in the beginning phase of treatment did not significantly predict subsequent depression scores at the end of treatment. General skills did significantly predict subsequent middle phase patient activation scores during the course of treatment and these activation scores significantly predicted reduced depression scores. A similar but nonsignificant trend was found for treatment-specific skills and patient-activation scores (*p* < 0.10). The indirect effect of general skills (but not treatment-specific skills) and patient-reported behavior activation on depression outcomes was significant; therefore, increased patient activation in the middle phase mediated the effects of better general skills in the early phase on improved depression outcomes at 3-months post-enrollment. There was no evidence of multicollinearity between any independent variables in the multiple regression models (VIF < 3).

**Table 1.** Regression and mediating analyses of mean depressive symptoms at 3-months (*N* = 50)

Predictors	*β*^a^	s.e.	Bootstrap 95% CI	Evidence for mediation
Model 1. Treatment-specific skills, patient activation and depressive (PHQ-9) symptoms^b^			(−1.456 to 0.162)	No
* c*′ (treatment-specific skills → PHQ-9)	−0.307(ns)	1.078		
* a* (treatment-specific skills → Patient activation)	1.401^‡^	0.493		
* b* (patient activation → PHQ-9)	−0.331*	0.184		
* a* × *b*	−0.464			
Model 1a. General skills, patient activation and depressive symptoms			(−5.811 to −0.142)	Yes
* c*′ (general skills → PHQ-9)	−0.886(ns)	1.607		
* a* (general skills → patient activation)	2.486*	1.191		
* b* (patient activation → PHQ-9)	−1.028***	0.276		
* a* × *b*	−2.555			

a Beta estimates are not standardized.

b Control variables included baseline PHQ-9 scores, health counselor, recruitment period, and where relevant, therapy quality scores.

^‡^*p*<0.10. *p<0.05. **p<0.01. ***p<0.001.

### Therapy quality, change talk and counter-change talk, and harmful drinking outcomes

The mean treatment-specific and general skills scores were 2.30 (95% CI 2.17–2.43) and 2.45 (95% CI 2.32–2.57) respectively in session 1 and mean frequency counts of change talk and counter-change scores were 16.05 (95% CI 13.92–18.17) and 14.97 (95% CI 12.85–17.11) respectively in session 2. Correlational analyses indicated nonsignificant trend-level or fully significant, associations between treatment-specific skills and patient change talk (*r* = −0.229, *p* = 0.095), general skills and counter-change talk (*r* = −0.229, *p* = 0.045), as well as a significant negative relation between counter-change-talk (*r* = 0.364, *p* = 0.009) and mean daily alcohol consumption outcome (*r* = 0.525, *p* = 0.0001). These, in turn, suggest small to large effect sizes between independent and dependent variables of interest (Cohen, 1988). Treatment-specific therapy quality skills were significantly related to general skills (*r* = 0.866, *p* < 0.001) whereas session-wise ratings of change and counter-change-talk were not (*r* = −0.080, *p* = 0.589). Patient education levels also were significantly related to one or more key variables and, along with baseline AUDIT scores and health counselor, were therefore added to the regression models.

**Fig. 2.**
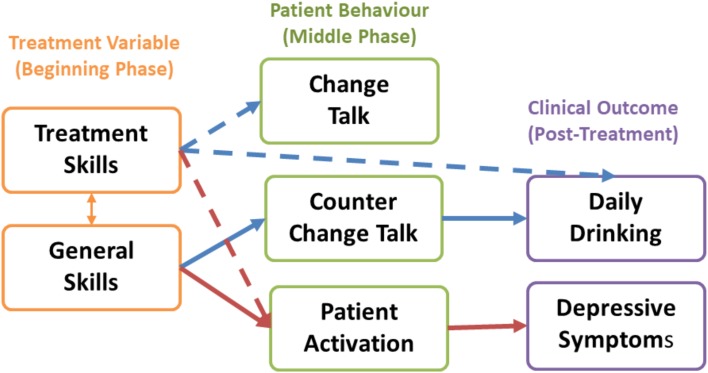
Predictive and mediating roles of therapy quality, patient behaviors, and clinical outcomes for depression and harmful drinking.

Table 2 and Fig. 2 show the results of the regression and mediation analyses. First, there was a direct association between higher treatment-specific skills and reduced 3-month alcohol consumption at the *p* < 0.10 level, but not between general skills and reduced 3-month alcohol consumption. Second, at non-significant levels, higher treatment-specific skills in the beginning phase of treatment were associated with increased change talk and reduced counter-change talk in the middle phase. We also found a significant relationship between higher general skills in the early phase and increased change talk in the middle phase, and a nonsignificant relation between general skills and counter-change talk in the middle phase. Third, lower counter-change talk, but not change-talk, significantly predicted reduced mean alcohol consumption at 3-months. Finally, the indirect effect of general therapy quality skills and counter-change talk on reduced drinking was significant, thus suggesting that lower counter-change talk mediated the effect of higher general skills on reduced harmful drinking at 3-months. There was no evidence of multicollinearity between any independent variables in any of the multiple regression models (VIF < 2).

**Table 2.** Regression and mediating analyses of mean daily alcohol consumption at 3-months (*N* = 50)

Predictors	*β*^a^	SE	Bootstrap 95% CI	Evidence for mediation
Model 1a. Treatment-specific skills, change talk, and alcohol consumption^b^			–	No
* c*′ (treatment-specific skills → alcohol consumption)	−22.023^‡^	13.012		
* a* (treatment-specific skills → change talk)	3.099^‡^	2.340		
* b* (change talk → alcohol consumption)	−0.421(ns)	0.919		
* a* × *b*	−68.251^c^			
Model 1b. Treatment-specific skills, counterchange talk, and alcohol consumption			(−59.59 to 1.361)	No
* c*′ (treatment-specific skills → alcohol consumption)	−22.023^‡^	13.012		
* a* (treatment-specific skills → counterchange talk)	−8.865^‡^	4.7439		
* b* (counterchange talk → alcohol consumption)	2.753***	0.767		
* a* × *b*	−25.293			
Model 2a. General skills, change talk, and alcohol consumption			–	No
* c*′ (general skills → alcohol consumption)	−11.732(ns)	15.296		
* a* (general-specific → change talk)	5.784*	2.606		
* b* (change talk → alcohol consumption)	−0.549(ns)	0.976		
* a* × *b*	−3.173^c^			
Model 2a. General skills, counterchange talk, and alcohol consumption			(−41.19 to −11.060)	Yes
* c*′ (general skills → alcohol consumption)	−11.732(ns)	15.296		
* a* (general-specific → counterchange talk)	−7.714^‡^	5.474		
* b* (counterchange talk → alcohol consumption)	3.178***	0.751		
* a* × *b*	−24.515			

a Beta estimates are not standardized.

b Control variables included baseline AUDIT scores, health counselor, and patient educational level, marital status and where relevant, therapy quality subscales.

c Mediation conditions were not met (i.e. no effect of M → Y) and therefore indirect effects were not tested for mediation.

^‡^*p*<0.10. *p<0.05. **p<0.01. ***p<0.001.

## Discussion

The aim of the current study was to explore the temporal pathway of change within two treatments, one for depression and the other for harmful drinking. The specific aim was to investigate the predictive and mediating roles of therapy quality, both general and specific, and patient behaviors on mean depressive symptoms and average daily alcohol consumption outcomes respectively at 3-months post enrollment.

### A temporal pathway for change to reduce depressive symptoms and harmful drinking

In line with our hypotheses, we observed preliminary evidence suggesting a temporal pathway in HAP in which therapy quality in the beginning phase of treatment worked through patient activation in the middle phase to reduce subsequent depressive symptoms. Similarly, we found that the effect of general skills was mediated through decreased counter-change talk to ultimately influence mean drinking outcomes. While MI-consistent behaviors have been linked to both change talk and counter-change talk, few have disentangled treatment-specific skills from general nonspecific skills. These findings are broadly consistent with the theories of change that provide the conceptual basis for each of the treatments (Jacobson *et al*., 2001 for HAP; Magill *et al*., 2018 for CAP).

#### General skills *v*. treatment skills

Higher levels of both treatment-specific and general skills were associated with positive effects on outcomes for both depression and harmful alcohol use. For HAP, higher levels of both sets of skills were associated with improved patient-activation levels, although only the effects of general skills on reduced depressive symptoms were mediated by patient activation. Importantly, this relation was significant when considering general skills but only evidenced a nonsignificant effect between treatment-specific and patient activation. These findings, if confirmed, highlight the importance of general common skills in determining the outcome of this PT. Similarly, for CAP, a nonsignificant trend was found between treatment-specific therapy quality skills and reduced harmful drinking. Importantly, only higher levels of general skills were mediated by decreased counter-change talk, and not change talk, in reducing drinking outcomes. These findings suggest that treatment-specific skills are contingent on general skills and that treatment-specific and general skills, while related with one another, are differentially related to both treatment outcome and patient behavior in independent ways.

#### Counter-change talk *v*. change talk

The CAP findings highlight the importance of reducing counter-change talk, rather than stimulating change talk (Magill *et al*., 2014; Romano and Peters, 2016), which the current analyses suggest ultimately influences patient drinking outcomes. While others have demonstrated the importance of counter-change talk, this has often been alongside change-talk for alcohol (e.g. Moyers *et al*., 2009). Our findings are consistent with research from the general psychotherapy and behavior change literature that shows that less counter-change talk in early sessions is a stronger predictor of positive outcomes than more change talk (Lombardi *et al*., 2014). The current findings are also consistent with two recent meta-analytic reviews of MI that found a robust role for counter-change talk but not change talk in relation to behavioral outcomes (Magill *et al*., 2014; Pace *et al*., 2017). As theorized in MI, change talk may operate only under specific conditions, with greater counselor experience and more severe alcohol problems (Gaume *et al*., 2013), both of which were limited in the current study context. For this reason, counter-change talk (aka ‘sustain’ talk) is more consistently related to treatment outcomes than change talk (Magill *et al*., 2018).

### Limitations and implications

There were three main limitations. First, the sample sizes were small. Independent and larger samples are needed to replicate and extend the current findings. Second, other treatment, therapist, and patient-level variables could have been assessed. For example, we did not measure therapeutic alliance from the patient perspective – a frequently studied phenomenon in the PT literature (Ackerman and Hilsenroth, 2003) – although one that garners mixed results as a potential mediator (e.g. Martin *et al*., 2000; Lemmens *et al*., 2016). In addition, we did not assess how cognitions may have influenced patient activation and outcomes. This has been examined in other trials (Lemmens *et al*., 2017) and the interplay of cognition and behavioral components may inform how brief PTs work. We also did not examine change or counter-change talk among HAP participants or patient activation among CAP patients. Third, in order to assess temporally distinct relations, we evaluated therapy quality only in the beginning phase of treatment and patient activation only during the middle phase of treatment. However, it is possible that they influenced one another in a reciprocal fashion and their relations over time remains unknown. Future studies need to consider the potential role and timing of multiple treatment and patient variable to expand on the temporal pathway highlighted here.

The main implication of our study is that maximizing therapy quality in early sessions is likely to mobilize patient behaviors that ultimately work to improve clinical outcomes. In HAP, our results support the BA model that highlights the central and mediating role of patient-reported activation in alleviating depressive symptoms. In CAP, our results expand on the temporal pathway model to highlight the central and mediating role of counter-change talk, but not change talk, in reducing daily alcohol consumption. These findings are particularly important when training NSPs in suggesting the role and monitoring of therapy quality on potential mediators to ultimately influence treatment effectiveness.

## Footnotes

† The notes appear after the main text.

## Notes

1 The HAP and CAP TQS were previously referred to as the Quality for Healthy Activity Program (Q-HAP) and Quality for Counselling for Alcohol Problems (Q-CAP) Scale, respectively, but were modified during the larger trial for consistency and ease of understanding.

2 Mediation conditions were met if the aforementioned regression models demonstrated that there were significant effects of the independent variable on the proposed mediator (X → M) and of the proposed mediator on outcome scores (M → Y; Mackinnon et al., 2007*a*, *b*), where significance was defined as *p* < 0.10 (Hayes, 2015).
